# Comparative Evaluation of Standard E TB-Feron ELISA and QuantiFERON-TB Gold Plus Assays in Patients with Tuberculosis and Healthcare Workers

**DOI:** 10.3390/diagnostics11091659

**Published:** 2021-09-10

**Authors:** In Young Yoo, Jaewoong Lee, Ae Ran Choi, Yoon Hee Jun, Hwa Young Lee, Ji Young Kang, Yeon-Joon Park

**Affiliations:** 1 Department of Laboratory Medicine, Seoul St. Mary’s Hospital, College of Medicine, The Catholic University of Korea, Seoul 06591, Korea; yiy00@naver.com (I.Y.Y.); 93031007@cmcnu.or.kr (A.R.C.); 2 Department of Laboratory Medicine, Incheon St. Mary’s Hospital, College of Medicine, The Catholic University of Korea, Incheon 21431, Korea; enemaz@hanmail.net; 3 Division of Infectious Disease, Department of Internal Medicine, Seoul St. Mary’s Hospital, College of Medicine, The Catholic University of Korea, Seoul 06591, Korea; koreajunmd@gmail.com; 4 Division of Allergy, Department of Internal Medicine, Seoul St. Mary’s Hospital, College of Medicine, The Catholic University of Korea, Seoul 06591, Korea; lehwyo@catholic.ac.kr; 5 Division of Pulmonary Medicine, Department of Internal Medicine, Seoul St. Mary’s Hospital, College of Medicine, The Catholic University of Korea, Seoul 06591, Korea; rkdwldud@catholic.ac.kr

**Keywords:** tuberculosis, healthcare workers, diabetes mellitus, borderline, QuantiFERON-TB Gold Plus

## Abstract

Recently, the American Thoracic Society/Infectious Diseases Society of America/Centers for Disease Control and Prevention advised against performing the interferon-γ-release assay (IGRA) test for individuals with a low risk of TB, and also recommended retesting low-risk individuals with an initial positive IGRA result. However, to evaluate both sensitivity and specificity of available tests, we compared the performance of the Standard E TB-Feron (TBF) and QuantiFERON-TB Gold Plus (QFT-Plus) assays in healthcare workers (HCWs) and tuberculosis (TB) patients. We also retrospectively investigated diabetes mellitus (DM) comorbidity among the enrolled TB patients. We prospectively collected samples from 177 HCWs and 48 TB patients. The TBF and QFT-Plus tests were performed and analyzed according to the manufacturers’ instructions. We also defined IGRA results between 0.2 and 0.7 IU/mL as ‘borderline’. The agreement rate between TBF and QFT-Plus was 92.0% (207/225) with a Cohen’s kappa value of 0.77 (95% CI, 0.68–0.87). While the majority (26/31, 83.9%) of borderline TBF results were in HCWs, the majority (14/19, 73.7%) of borderline QFT-Plus results were in TB patients. Discordant results were found in 18 samples, with TBF-positive/QFT-Plus-negative or indeterminate results in 11 HCWs and seven TB patients. After resampling from 10 HCWs (seven borderline-positive and three positive results, all <1.0), six reverted to negative. The prevalence of DM comorbidity was very high (35.4%). In summary, TBF showed substantial agreement with the QFT-Plus assay but had a higher positivity rate in both HCWs and TB patients. The negative conversion rate was high (60%) among HCWs whose initial (TB Ag-nil) result was <1.0.

## 1. Introduction

Tuberculosis (TB) is a global health problem, with 10.0 million new cases of TB and 1.2 million TB deaths among non-HIV patients in 2019 [1]. Although the incidence of TB has been decreasing for decades, the incidence of diabetes mellitus (DM), which increases the risk of developing TB by 2–3-fold and also increases the risk of TB treatment failure, relapse, and death, is increasing worldwide [2]. Notably, sustained hyperglycemia (HbA1c ≥ 7.0%) and pre-diabetes mellitus (HbA1c 5.7–6.4%) have been associated with increased TB prevalence [3]. Korea still has a high TB incidence rate, which was 51 per 100,000 people in 2018, and also has a high prevalence (14.4% in 2016) of DM among adults aged 30 years and above [4].

An immunoassay that measures the interferon (IFN)-γ response to Mycobacterium tuberculosis-specific antigens (interferon-γ-release assay; IGRA) has been used to diagnose latent tuberculosis infection (LTBI) [1]. Although IGRA is more specific for M. tuberculosis infection than the tuberculin skin test (TST) [5], several factors, such as old age, body mass index < 16.0 kg/m^2^, HIV co-infection, and homozygosity for HLA-DRB1*0701, are known to lower the sensitivity of IGRAs [6]. In addition, DM is associated with insufficient immune responses due to the impaired performance of immune cells [7]. As a result of this impaired immune cell performance and insufficient IFN-γ response [7], the sensitivity of IGRAs is lower than that of the TST in DM patients with smear-negative tuberculosis [8]. At present, three FDA-cleared IGRAs are commercially used: (1) T-SPOT.TB (Oxford Immunotec, Abingdon, UK), (2) QuantiFERON-TB Gold In-Tube (QFT-GIT; Qiagen, Hilden, Germany), and (3) QuantiFERON-TB Gold Plus (QFT-Plus; Qiagen), which is a new generation of QFT-GIT. QFT-GIT contains a TB antigen tube, whereas QFT-Plus contains two TB antigen tubes (TB1 and TB2): the TB1 antigen tube contains long peptides derived from ESAT-6 and CFP-10 (TB 7.7, which was included in the previous QFT-GIT version, has been removed) and is designed to induce a specific CD4 T-cell response; the TB2 antigen tube contains not only the long peptides of TB1 but also shorter peptides from ESAT-6 and CFP-10 to detect both CD4 and CD8 T-cell responses.

Recently, the Standard E TB-Feron enzyme-linked immunosorbent assay (ELISA) (TBF; SD Biosensor, Gyeonggi-do, Republic of Korea) was CE marked and approved by the Ministry of Food and Drug Safety of the Republic of Korea for the diagnosis of latent TB. The TB antigen tube contains whole recombinant proteins of ESAT-6, CFP-10, and TB7.7. The use of whole proteins instead of peptides increases the sensitivity of the assay because when the proteins are degraded into diverse small peptides, multiple epitopes may be present; thus, stimulating T cells more effectively and potentially yielding higher IFN-γ values [9]. Although both IGRA tests are approved for the diagnosis of latent TB, given the complexities involved in diagnosing this illness, we evaluated the performance of TBF compared with QFT-Plus not only in healthcare workers (HCWs) but also in confirmed TB patients. In addition, we retrospectively investigated DM comorbidity among the enrolled TB patients.

## 2. Materials and Methods

### 2.1. Study Population

From January to September 2020, we prospectively and consecutively enrolled participants among HCWs and active TB patients who visited the department of pulmonary medicine for the treatment of TB. All participants were adults aged above 18 years. Active TB was diagnosed by isolation of the M. tuberculosis complex by culture and/or positive results from the AdvanSure TB/NTM real-time PCR kit (LG Life Sciences, Seoul, Korea) or Xpert MTB/RIF (Cepheid, Sunnyvale, CA, USA). Prior to the study, HCWs were confirmed to have negative IGRA results using QFT-GIT. The risk of TB exposure in HCWs was investigated via a questionnaire survey about previous TB history and personal contact with TB patients. This study was approved by the Institutional Review Board (IRB) of Seoul St. Mary’s Hospital, Catholic University of Korea (IRB No. KC19DESI0580), and written informed consent was obtained from all participants meeting enrollment criteria.

### 2.2. Standard E TB-Feron ELISA and QuantiFERON-TB Gold Plus Assays

Both assays were performed according to the manufacturers’ instructions. The complete procedures were identical between the two tests except that QFT-Plus included two TB antigen tubes (TB1 and TB2). Whole blood was collected in heparinized vacutainer tubes (BD, Franklin Lakes, NJ, USA) in the following order: nil, TB antigen (TB1 and TB2 tubes for QFT-Plus), and mitogen. After mixing, the tubes were placed into a 37 °C incubator for 16–20 h. Then, the tubes were centrifuged for 15 min at 2500× *g*, and the separated plasma was stored at −20 °C until performing ELISA assays. The TBF and QFT-Plus ELISA assays were performed on an Evolis™ (Bio-Rad Laboratories, Inc., Hercules, CA, USA) and Dynex DS2^®^ automated ELISA analyzer (Dynex Technologies, Chantilly, VA, USA), respectively. The results were interpreted according to the manufacturers’ instructions, in which the criteria for positivity/negativity and indeterminate were identical; a positive result was defined as an IFN-γ value for the TB antigen (TB1 or TB2 for QFT-Plus)–nil of ≥0.35 IU/mL and ≥25% of the nil value, and negative was defined as TB antigen–nil  < 0.35  IU/mL or  <25% of the nil value when mitogen  ≥  0.5  IU/mL. Results not meeting any of these criteria were considered indeterminate. However, as several investigators have suggested a more rigorous interpretation of the QFT-Plus conversion definition (an increase from IFN-γ < 0.2 to >0.7 IU/mL) [10,11], we also defined IGRA results between 0.2 and 0.7 IU/mL as ‘borderline’. Therefore, all results were interpreted as positive (≥0.7), borderline-positive (≤0.35–<0.7), borderline-negative (≤0.2–<0.35), or negative (<0.2). Because QFT-Plus had TB1 and TB2 tubes, we selected the higher value between TB1 and TB2 to define the ‘borderline’ result; in other words, if one of the two tubes produced a value of >0.7, it was defined as ‘positive’; IFN-γ values in the antigen tubes >10 IU/mL were designated as 10 IU/mL. For individuals with discrepant results between the two assays, resampling was performed when possible.

### 2.3. Statistical Analysis

Data were analyzed using Microsoft Excel 2016 (Microsoft Corporation, Redmond, WA, USA) and SPSS software version 24.0 (IBM Corp., Armonk, NY, USA). The qualitative concordance between TBF and QFT-Plus was measured using Cohen’s kappa value; values of <0.21, 0.21–0.40, 0.41–0.60, 0.61–0.80, and 0.81–1.00 were interpreted as poor, fair, moderate, substantial, and almost perfect agreement, respectively.

Differences in frequencies were evaluated by Fisher’s exact test. For quantitative analysis, medians and interquartile ranges (IQRs) were calculated for continuous measures. The differences between continuous variables were analyzed using the Wilcoxon signed-rank test for paired comparisons. A *p*-value of <0.05 was considered statistically significant. IFN-γ values were compared between TBF and QFT-Plus by Spearman’s rank correlation and Passing-Bablok regression: Spearman’s rho (rs) values > 0.7 indicate high correlation, 0.7 ≥ rs > 0.5 indicates moderate correlation, and rs ≤ 0.5 indicates low correlation.

## 3. Results

Concordance between TBF and QFT-Plus is presented in Table 1. In total, the agreement rate between TBF and QFT-Plus was 92.0% (207/225) with a Cohen’s kappa value of 0.77 (95% CI, 0.68–0.87). The overall/positive/negative agreement rates in HCWs and active TB patients were 93.8%/100%/93.8% and 85.4%/100%/not available, respectively.

In Table 2, the positive, negative, borderline-positive, and borderline-negative results among the 177 HCWs and 48 TB patients are presented. Among the 177 HCWs, QFT-Plus showed 172 negative results and 5 borderline results (4 borderline-negative and 1 borderline-positive), whereas TBF showed 148 negative results, 3 positive results, and 26 borderline results (9 borderline-positive and 17 borderline-negative results). When performed on the 177 HCWs, the proportion of negative results showed a statistically significant difference between the two assays (172/177 for QFT-Plus vs. 148/177 for TBF, *p*-value < 0.05). Based on the responses to the questionnaire by 172 HCWs, 66 had a history of TB exposure within the previous 2 years, and 7 of them showed positive or borderline-positive results. In contrast, only 5 out of the 106 participants without a history of TB exposure showed positive or borderline-positive results (*p*-value 0.08). However, although the number of cases was very small, negative conversion was observed in 5 out of 7 HCWs (71.4%) who had been exposed to TB, while it was observed in 1 of 3 HCWs (33.3%) without TB exposure (*p*-value 0.26).

Among the 48 TB patients, QFT-Plus showed 36 positive results, 10 borderline results (5 borderline-positive and 5 borderline-negative), and 1 negative and 1 indeterminate result. With TBF, 43 positive results and 5 borderline-positive results were obtained.

The results of 18 samples were discordant between the two assays: 17 were TBF-positive/QFT-Plus-negative, and 1 was TBF-positive/QFT-Plus indeterminate. The discordant results were from 11 HCWs and 7 TB patients (Table 3 and Table 4). Among the 18 cases, resampling was performed for 10 HCWs approximately one year later, and re-testing revealed that none of the 7 HCWs who had borderline-positive results showed positive conversion. In contrast, among the three HCWs who showed initial results of 0.80, 0.95, and 0.72, two of them had positive results when retested.

Retrospective chart review showed that, among the 48 TB patients, 17 (35.4%) had DM. The false-negative rate of QFT-plus was slightly higher in TB patients with DM than without DM (17.6% (3/17) vs. 12.9% (4/31), respectively), but it was not statistically significant (*p*-value 0.69).

## 4. Discussion

In general, the two tests showed a high concordance rate (92.0%), but TBF had a higher positivity rate than QFT-Plus. In addition, many of the borderline-positive results converted to negative in the re-examination. Of the 177 HCWs, 11 had discrepant results (TBF-positive/QFT-Plus-negative), although eight of them were ‘borderline’ positive. After testing the re-collected specimens from HCWs, five (62.5%) of the eight borderline-positive results reverted to negative (<0.35 IU/mL). This finding is in line with a German study, in which serial testing in individuals whose initial results were close to the cut-off value showed a high reversion rate (6/18, 33%) [12]. In addition, Metcalfe et al. reported that the normal expected range of within-subject variability for QFT-GIT was ±0.24 IU/mL among subjects with a borderline TB response (0.25–0.80 IU/mL) [13]. The cause of the high reversion rate in the borderline range is not clear, but possible sources include various factors (e.g., collected blood volume, tube shaking, tube order, incubation and processing delay, and immune modulation by microbial products) of the test procedure [14,15] and within-subject variability [13]. Recently, this limitation was acknowledged by the American Thoracic Society/Infectious Diseases Society of America/Centers for Disease Control and Prevention, and the recent guidelines advise against testing for LTBI in individuals who are unlikely to be infected with M. tuberculosis. The guidelines also recommend retesting low-risk individuals with an initial positive IGRA result, with a negative result in the retest overriding the initial positive result [16].

Notably, of the 48 TB patients, seven patients had negative/borderline-negative/indeterminate results with QFT-Plus, whereas all of the patients had either positive (43 patients) or borderline-positive results (five patients) with TBF. While a few borderline results (5/31, 16.1%) of TBF were in TB patients, the majority (10/15, 73.7%) of the borderline results of QFT-Plus were in TB patients (*p*-value < 0.05). Among the above seven patients, three were comorbid for DM. This is consistent with a previous study [17], in which two out of three active TB patients with negative or indeterminate results in both QFT-Plus and QFT-GIT were comorbid for DM. In addition, Choi et al. reported that reduced sensitivity of QFT-Plus was associated with sputum smear-negative TB patients with DM [7]. To overcome this limitation, considering that TB-specific CD8+ T lymphocytes are more efficiently stimulated by the recombinant protein antigens used in TBF, TBF would be a useful alternative to current IGRAs such as QFT-Plus, especially for immunocompromised patients who undergo IGRA tests to diagnose MTB infection [18].

In addition, as individuals with DM are more prone to TB infection and treatment failure, bidirectional surveillance can help to diagnose and treat both diseases. Further study is needed to evaluate the usefulness of IGRA tests for the early diagnosis of latent TB in DM patients.

## Figures and Tables

**Table 1:** Concordance between Standard E TB-Feron ELISA and QuantiFERON-TB Gold Plus assays.

		QFT-Plus Result				% Agreement (95% CI)			Kappa Value (95% CI)
	TBF Result	Positive	Negative	Indeterminate	Total	Overall Percent Agreement	Positive Percent Agreement	Negative Percent Agreement	
Total	Positive	42	17	1	60	92.0% (87.5–95.1%)	100% (89.6–100%)	90.2% (84.7–93.9%)	0.77 (0.68–0.87)
(*n* = 225)	Negative	0	165	0	165				
	Total	42	182	1	225				
HCW	Positive	1	11	0	12	93.8% (88.9–96.7%)	100% (5.0–100%)	93.8% (88.8–96.7%)	0.14 (0.0–0.40)
(*n* = 177)	Negative	0	165	0	165				
	Total	1	176	0	177				
TB patient	Positive	41	6	1	48	85.4% (71.6–93.5%)	100% (51.7–100%)	Not available	Not available
(*n* = 48)	Negative	0	0	0	0				
	Total	41	6	1	48				

TBF, Standard E TB-Feron ELISA; QFT-Plus, QuantiFERON-TB Gold Plus assay; CI, confidence interval.

**Table 2:** Distribution of 225 samples with respect to the borderline range (0.20–0.70 IU/mL) in Standard E TB-Feron ELISA and QuantiFERON-TB Gold Plus assays.

	Standard E TB-Feron ELISA				QuantiFERON-TB Gold Plus Assay				
	Borderline (0.20–0.70)		Non-Borderline		Borderline (0.20–0.70)		Non-Borderline		
No. of Samples	Negative (0.20–0.35)	Positive (0.35–0.70)	Negative (<0.20)	Positive (>0.70)	Negative ^1^ (0.20–0.35)	Positive (0.35–0.70)	Negative (<0.20)	Indeterminate	Positive (>0.70)
HCWs (*n* = 177)	17	9	148	3	4	1	172	0	0
TB Patients (*n* = 48)	0	5	0	43	5	5	1	1	36

HCW, healthcare worker; TB, tuberculosis. ^1^ IFN-γ value of TB1 or TB2 antigen for QFT-Plus–nil within uncertainty zone.

**Table 3:** IFN-γ values and active TB exposure history for 11 HCWs with discordant results between Standard E TB-Feron ELISA (positive) and QuantiFERON-TB Gold Plus assays (negative).

No.	Standard E TB-Feron ELISA				Previous Active TB Treatment History	TB Exposure History of within 2 Years
	Primary Test		Resampling Test			
	TB–nil (IU/mL)	Result	TB–nil (IU/mL)	Result		
1	0.40	Positive	0.24	Negative	No	Yes
2	0.35	Positive	0.36	Positive	No	No
3	0.80	Positive	0.22	Negative	No	Yes
4	0.39	Positive	0.20	Negative	No	No
5	0.95	Positive	0.94	Positive	No	Yes
6	0.43	Positive	0.32	Negative	No	Yes
7	0.58	Positive	0.03	Negative	No	Yes
8	0.72	Positive	0.74	Positive	No	No
9	0.39	Positive	0.10	Negative	No	Yes
10	0.39	Positive	0.46	Positive	No	Yes
11	0.68	Positive	ND		No	No

HCW, healthcare worker; ND, not done.

**Table 4:** Characteristics of seven TB patients with discordant results (Standard E TB-Feron ELISA (positive) and QuantiFERON-TB Gold Plus assay (negative)).

No.	Age (year)/Sex	Diagnosis of TB	Treated with Immunosuppressive Drugs	Underlying Disease	Standard E TB-Feron ELISA	
					TB–nil (IU/mL)	Result
1	53/M	Pulmonary TB	No	None	0.78	Positive
2	49/F	Pulmonary TB	No	None	0.42	Positive
3	76/M	Pulmonary TB	No	CKD, DM, HTN	≥10	Positive
4	63/M	Pulmonary TB	No	None	0.43	Positive
5	76/M	Pulmonary TB	No	HTN	5.83	Positive
6	76/M	TB abscess on Rt. humerus	Yes (Tacrolimus)	DM, HTN, KT	1.17	Positive
7	61/M	Pulmonary TB	No	DM, Hyperlipidemia	0.79	Positive

M, male; F, female; TB, tuberculosis; CKD, chronic kidney disease; DM, diabetes mellitus; HTN, hypertension; KT, kidney transplantation.

## Data Availability

The data presented in this study are available on request from the corresponding author. The data are not publicly available due to privacy.

## References

[1] WHO (2020). Global Tuberculosis Report 2020.

[2] Badawi A., Sayegh S., Sallam M., Sadoun E., Al-Thani M., Alam M.W., Arora P. (2014). The Global Relationship between the Prevalence of Diabetes Mellitus and Incidence of Tuberculosis: 2000–2012. Glob. J. Health Sci..

[3] Al-Rifai R.H., Pearson F., Critchley J., Abu-Raddad L.J. (2017). Association between diabetes mellitus and active tuberculosis: A systematic review and meta-analysis. PLoS ONE.

[4] Kim B.-Y., Won J.C., Lee J.H., Kim H.-S., Park J.H., Ha K.H., Won K.C., Kim D.J., Park K.S. (2019). Diabetes Fact Sheets in Korea, 2018: An Appraisal of Current Status. Diabetes Metab. J..

[5] Sollai S., Galli L., de Martino M., Chiappini E. (2014). Systematic review and meta-analysis on the utility of Interferon-gamma release assays for the diagnosis of *Mycobacterium tuberculosis* infection in children: A 2013 update. BMC Infect. Dis..

[6] Le Hang N.T., Lien L.T., Kobayashi N., Shimbo T., Sakurada S., Thuong P.H., Hong L.T., Tam D.B., Hijikata M., Matsushita I. (2011). Analysis of Factors Lowering Sensitivity of Interferon-γ Release Assay for Tuberculosis. PLoS ONE.

[7] Faurholt-Jepsen D., Aabye M.G., Jensen A.V., Range N., PrayGod G., Jeremiah K., Changalucha J., Faurholt-Jepsen M., Jensen L., Jensen S.M. (2014). Diabetes is associated with lower tuberculosis antigen-specific interferon gamma release in Tanzanian tuberculosis patients and non-tuberculosis controls. Scand. J. Infect. Dis..

[8] Choi J.C., Jarlsberg L.G., Grinsdale J.A., Osmond D.H., Higashi J., Hopewell P.C., Kato-Maeda M. (2015). Reduced sensitivity of the QuantiFERON® test in diabetic patients with smear-negative tuberculosis. Int. J. Tuberc. Lung Dis..

[9] Kweon O.J., Lim Y.K., Kim H.R., Kim T.-H., Lee M.-K. (2019). Evaluation of Standard E TB-Feron Enzyme-Linked Immunosorbent Assay for Diagnosis of Latent Tuberculosis Infection in Health Care Workers. J. Clin. Microbiol..

[10] van Zyl-Smit R., Pai M., Peprah K., Meldau R., Kieck J., Juritz J., Badri M., Zumla P.S.A., Sechi L.A., Bateman E.D. (2009). Within-Subject Variability and Boosting of T-Cell Interferon-γ Responses after Tuberculin Skin Testing. Am. J. Respir. Crit. Care Med..

[11] Nemes E., Rozot V., Geldenhuys H., Bilek N., Mabwe S., Abrahams D., Makhethe L., Erasmus M., Keyser A., Toefy A. (2017). Optimization and Interpretation of Serial QuantiFERON Testing to Measure Acquisition of Mycobacterium tuberculosis Infection. Am. J. Respir. Crit. Care Med..

[12] Zwerling A., Hof S.V.D., Scholten J., Cobelens F., Menzies D., Pai M. (2011). Interferon-gamma release assays for tuberculosis screening of healthcare workers: A systematic review. Thorax.

[13] Metcalfe J.Z., Cattamanchi A., McCulloch C.E., Lew J.D., Ha N.P., Graviss E.A. (2013). Test Variability of the QuantiFERON-TB Gold In-Tube Assay in Clinical Practice. Am. J. Respir. Crit. Care Med..

[14] Banaei N., Gaur R.L., Pai M. (2016). Interferon Gamma Release Assays for Latent Tuberculosis: What Are the Sources of Variability?. J. Clin. Microbiol..

[15] Ringshausen F.C., Nienhaus A., Costa J.T., Knoop H., Schlösser S., Schultze-Werninghaus G., Rohde G. (2011). Within-Subject Variability of *Mycobacterium tuberculosis*-Specific Gamma Interferon Responses in German Health Care Workers. Clin. Vaccine Immunol..

[16] Lewinsohn D.M., Leonard M.K., LoBue P.A., Cohn D.L., Daley C.L., Desmond E., Keane J., Lewinsohn D.A., Loeffler A.M., Mazurek G.H. (2017). Official American Thoracic Society/Infectious Diseases Society of America/Centers for Disease Control and Prevention Clinical Practice Guidelines: Diagnosis of Tuberculosis in Adults and Children. Clin. Infect. Dis..

[17] Theel E.S., Hilgart H., Breen-Lyles M., McCoy K., Flury R., Breeher L.E., Wilson J., Sia I.G., Whitaker J.A., Clain J. (2018). Comparison of the QuantiFERON-TB Gold Plus and QuantiFERON-TB Gold In-Tube Interferon Gamma Release Assays in Patients at Risk for Tuberculosis and in Health Care Workers. J. Clin. Microbiol..

[18] Jung J., Jhun B., Jeong M., Yoon S., Huh H., Jung C., Kim K., Park J., Kim D., Huh W. (2021). Is the New Interferon-Gamma Releasing Assay Beneficial for the Diagnosis of Latent and Active *Mycobacterium tuberculosis* Infections in Tertiary Care Setting?. J. Clin. Med..

